# Metastatic cells: moving onco-targets

**DOI:** 10.18632/oncotarget.2057

**Published:** 2014-05-31

**Authors:** Antonia Patsialou, John S. Condeelis

**Affiliations:** Research Department of Cancer Biology, UCL Cancer Institute, University College London, London, UK.; Department of Anatomy and Structural Biology, Gruss Lipper Biophotonics Center, Albert Einstein College of Medicine, Bronx NY, USA.

Breast cancer is the most frequent malignant neoplasm occurring in women, and metastasis of breast cancer is the main cause of death in these patients [1]. Metastasis is a complicated, multi-step process that is still poorly understood. In reality, for most patients, we just do not know whether they will develop metastasis, and therefore, as a precaution patients are overtreated. In the last decade, advances in genomic profiling attempted to fill in this gap: to predict which patients are at a higher risk of metastasis, therefore sparing the rest from the unnecessary harm of chemotherapy. In such studies, whole tumor tissue was analyzed, and the gene expression profiles of tumors from patients that developed metastasis were compared to those that did not. Such signatures are prognostic of metastatic relapse, and have led to the first FDA-approved microarray-based diagnostic test (Mammaprint) [2]. However, most of these signatures are composed mainly of cell cycle genes, and therefore there is a growing concern in the scientific community about whether they truly add information to the standard clinicopathological parameters of receptor status and proliferation [3]. In addition, they give us little information about the biological mechanisms of metastasis.

**Figure 1:**
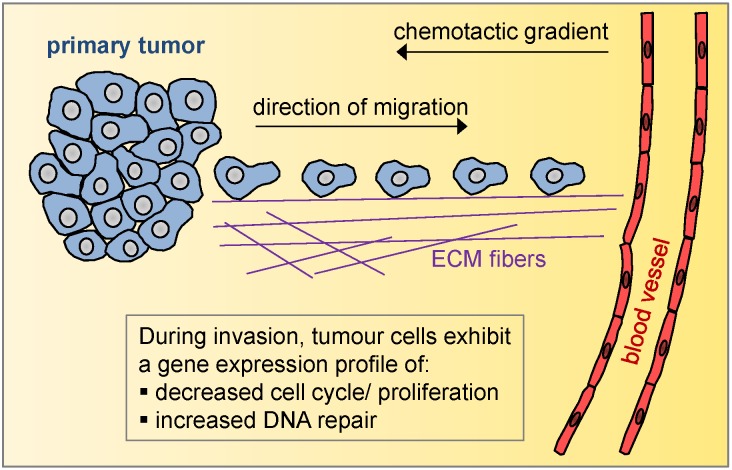
Main properties of invasive breast tumor cells as evident from the *in vitro* and the *in vivo* invasion signatures.

Cell migration and invasion are the early crucial steps of the metastatic cascade, where a small number of cancer cells inside the primary tumor invade the surrounding tissue and migrate towards chemotactic gradients leading to blood or lymphatic vessels (Figure 1) [4]. Due to the transient, rare and short-lived nature of this process, gene expression changes regulating invasion would be missed by profiling whole tissue chunks. It is therefore necessary to experimentally isolate the migratory cells in order to successfully analyze their contribution to tumor progression. In a study published recently by Limame *et al*., migrated and invaded breast cancer cells were experimentally isolated from their non-motile counterparts *in vitro* by selectively profiling cells at the top and bottom of transwells where cells were permitted to move towards a chemotactic gradient [5]. The authors reported a migration signature resulting from cells moving unobstructed through the pores of non-coated transwells, as well as an invasion signature resulting from cells moving through matrigel-coated transwells. In order to represent more states of this transient process, the authors additionally profiled both early and late timepoints for each migration and invasion processes. All four resulting signatures, independently of the evaluated timepoint, were successful in predicting distant metastasis-free survival in published breast cancer cohorts. Finally, the authors identified KLF9, a gene downregulated in their signatures, as a potential novel metastasis suppressor; KLF9 overexpression changed cell morphology in MDA-MB-231 metastatic breast cancer cells to a less elongated and more epithelial phenotype, as well as abrogated their invasion properties *in vitro*.

Our group has also recently published an *in vivo* invasion signature, derived from isolating the live migratory breast cancer cells directly from primary tumors, and then comparing them to the general cancer cell population from the same tumors [6]. Although the MDA-MB-231 breast cancer cell line was used in both studies, the resulting gene lists did not completely overlap. A direct comparison shows that the *in vivo* invasion signature most resembles the *in vitro* “early invasion” signature, with a 26% gene overlap. A significant implication of this is that a portion of gene expression changes occurring during *in vivo* invasion are cell autonomous and can be replicated in *in vitro* systems. However, the biggest portion of the *in vivo* invasion signature is unique, suggesting that these gene expression changes are triggered directly by the tumor microenvironment. Importantly, although individual genes were not entirely identical in the two studies, pathway analyses showed similar pathways upregulated in both *in vitro* and *in vivo* invasion (Figure 1). First, cell cycle and proliferation are downregulated in both *in vitro* and *in vivo* invasion. This suggests that cancer cells transiently shut down their cell cycle while moving, probably due to the incompatibility of using their cytoskeleton in migration and cell division concurrently. Second, DNA repair pathways are upregulated in both *in vitro* and *in vivo* invasion. It is interesting to note that in neither system cells were treated with any DNA damaging agents, and therefore the process of invasion towards a chemotactic gradient in a short time period alone was sufficient to cause an upregulation of DNA repair genes. Our lab is investigating further the significance of these findings and how these may be linked to therapy resistance in invaded and disseminated tumor cells.

Overall, by experimentally isolating actively migrating tumor cells, Limame *et al.* as well as our group have been able to selectively interrogate invasive tumor cells and derive gene expression profiles specific to migration and invasion. Our studies have uncovered novel genes and biological pathways that govern these early steps of the metastatic cascade in breast cancer, and could potentially aid in patient risk stratification more efficiently than first generation whole tissue signatures.
